# It takes a community to train a future physician: social support experienced by medical students during a community-engaged longitudinal integrated clerkship

**Published:** 2019-07-24

**Authors:** Timothy Dubé, Robert Schinke, Roger Strasser

**Affiliations:** 1 Centre for Medical Education, McGill University, Québec, Canada; 2 School of Human Kinetics, Laurentian University, Ontario, Canada; 3 Northern Ontario School of Medicine, Sudbury and Thunder Bay, Ontario, Canada

## Abstract

**Background:**

Social support may be beneficial for medical students who must develop adaptive strategies to respond to the demands and challenges during third-year clerkship. We provide a detailed description of the supportive behaviours experienced by third-year students during a longitudinal integrated clerkship (LIC) in the context of rural family medicine.

**Methods:**

Informed by a social constructivist research paradigm, we undertook a qualitative study to understand from the students’ perspectives the presence and characteristics of social support available during a LIC. Data were collected from conversational interviews at three points during the eight-month clerkship year, pre-, during, and post-clerkship, to explore how 12 medical students experienced social support. We employed an innovative methodological approach, the guided walk method, to gain the students’ stories in the contexts where they were taking place.

**Results:**

The participants described the relationships they developed with various sources of social support such as (a) preceptors, (b) peers, (c) family, (d) health professionals, and (e) community members.

**Conclusion:**

Various individuals representing communities of practice such as the medical profession and community members were intimately related to the longitudinal aspects of the students’ experiences. The findings lend credence to the view that *it really does take a community to train a future physician*.

## Introduction

Social support contributes to general health and personal adjustment.^1,2^ Researchers concluded that the basis for social support lies in individuals’ views that they have close relationships with people who are willing to help them in times of need.^2,3^ Social support can be particularly beneficial for medical students who must develop adaptive strategies to respond to the demands and challenges of medical training.^4-6^ A critical transitional period in undergraduate medical education occurs during the third-year clerkship when students transition from primarily classroom learning to spending more time in clinical contexts,^7-13^ and when students can benefit from supportive behaviours by others such as preceptors, peers, family and friends.^6,14-16^ There is a great deal of pressure on the students to perform academically and clinically throughout their clerkship. For example, students must adapt to different learning and healthcare environments and must manage several challenges, including establishing new relationships with clinical teachers, adjustment to clinical practice, and the professional socialization of becoming a physician.^17-21^ There are also cultural expectations within the medical profession that urge students to learn to develop strategies to cope with these experiences and emotions independently.^7,21-23^ Anxiety, depression, substance abuse, mal- adjustment, and burnout are a few outcomes that can result when students lack adaptive strategies such as seeking out social support to manage the demands and challenges during their clerkship year.^4,5,24-26^

The perceived influence the preceptors have on students is double-edged. First, they have the potential to be a tremendous source of support for a medical student. Epstein et al.^15^ identified that students felt largely supported by their preceptors’ abilities to provide safe learning environments and bring forth positive teaching behaviours. Students in community-based contexts described feeling supported by their preceptors’ abilities to use strategies that promote effective teaching including coaching, mentorship, and encouraging reflection about their learning needs.^15,27^ However, the interpersonal dynamics with preceptors can also become negative, distracting, and have the potential to have a profoundly negative effect on students’ learning experiences.^8,9^ Researchers have found that students felt at times discouraged,^12^ humiliated, and embarrassed^28^ by their preceptors, and thus determined that social interactions with preceptors can be a significant source of stress rather than a source of support.

The camaraderie amongst peers can help to build relationships, augment social support (e.g., shared social reality touchstones - others sharing similar situational contexts who can help work through challenges),^14,29^ and reduce the deleterious effects of burnout.^5,24^ Bonney and colleagues^29^ described students’ sense of belonging to the healthcare teams and community contexts during a longitudinal integrated clerkship (LIC). They concluded that students’ perspectives regarding the success of their placement experience were largely influenced by the positive or negative interactions between environments (e.g., clinical, learning, community, social) and relationships with individuals (e.g., preceptors, healthcare team). They also found that students’ levels of engagement in the community were dependent upon communities promoting a sense of belonging and providing positive learning experiences.

Throughout their clerkships, students must negotiate many simultaneous identities such as student, community member, and professional. They must also determine whether, and how, they might participate in the social, physical, and medical community in which their clerkship takes place. LIC models differ from traditional clerkship models, where students learn in urban teaching hospitals during block rotations and come to know new people and new locations every four to six weeks.^30^ Furthermore, little is known from whom the students seek support and the integral role these individuals have when students are experiencing a LIC. We address this gap by describing the supportive behaviours experienced by third-year students during a LIC in the general context of rural family medicine. The purpose of our study was to learn which sources of social support students sought out during a rural- based LIC.

The LIC at the Northern Ontario School of Medicine (NOSM) in Canada consists of all students spending eight months in one of 15 rural and northern communities where they live, learn, and work during their clerkship. At least two students are placed in a community to facilitate a supportive learning environment. Students have concurrent exposure to the various clinical disciplines and aspects of medicine across the life cycle. NOSM’s LIC provides students with opportunities for continuity with clinicians in rural-based primary care settings. Students also gain insight into the healthcare experiences of patients through longitudinal and community-engaged learning. We published previously about the adaptive strategies that medical students employed during the stages in the transition processes throughout their LIC at NOSM.^8^ These stages consisted of going from learning in the classroom to learning in clinical contexts, the disorientation dilemma felt two to three months into the clerkship, and the professional socialization of seeing themselves as becoming physicians by the end of their clerkship. Getting to the core of the matter is the need to learn from the students’ vantages about the presence, nature, and characteristics of the relationships with the sources of social support they seek out. Therefore, our purpose was to answer the following research question: Which, if any, sources of social support do students experience during a LIC in the context of rural family practice?

## Methods

For social constructivists, learning is a process influenced socially and culturally by the environments in which individuals are surrounded.^31,32^ Informed by this research paradigm, we undertook a qualitative study through conversational interviews with 12 participants at three points in 2011/2012, pre-, during, and post-clerkship, including an innovative qualitative method during the clerkship, the guided walk. Guided walks place participants, contexts, and researchers centrally within the research process to facilitate meaningful representations of participants’ lived experiences.^33-36^ Data reported in this paper relate to the social support experienced by students throughout their LICs. Our study received research ethics approval from Laurentian University and Lakehead University.

### Participants

Participant recruitment began with a formal presentation of the study to the entire third-year class (56 students) during orientation week which occurred three months prior to the start of clerkship. Purposive and snowball sampling were used for participant recruitment. The key features of these approaches are based on the researcher’s accessibility to the participants and the notion of asking initial participants to direct the researcher to other potential participants. Twelve students agreed to take part in the study (21% of the student cohort), two men and 10 women. The mean age was 28.4 years. Table 1 provides detailed demographic information about the participants.

**Table 1:** Participant demographic information

Demographic characteristics	Demographic information (n=12)
Home province	11 from Ontario
Educational Background	Health sciences (e.g. nursing), Medical sciences (e.g. biochemistry), Social sciences, Arts
Marital status	6 married or in a civil arrangement
Children	1 with children

### Data collection

Two recent graduates of the NOSM’s MD Program were key informants throughout the research process. Their prior experience and knowledge in the particular context of the clerkship played a key role in the methodological approach. They described the significance of interviewing the students prior to the clerkship in order to understand their anticipations, at three months into the clerkship since they recalled that stage as a time when they experienced increased confidence and competence, and after the clerkship to appreciate how the students consolidated their learning. The key informants also emphasized the importance of visiting the students in their clerkship communities for the interviews at three months to situate their experiences in the contexts where they were taking place.

The pre-clerkship interviews occurred one month prior to the start of students’ placement. Guided by the participants and the flow of the conversation, the interviews ranged between 30 and 65 minutes. Additionally, participants were probed to ensure in- depth understanding and detailed descriptions of their lived experiences.

At three months into the clerkship, data were collected using a guided walk (lasting between one to two hours), which is an effective and innovative qualitative method where the participant walks (i.e., leads) the researcher through contexts felt to most significantly represent their lived experiences while engaged in a conversational interview.^35-37^ The duration of the walks and the routes taken were determined by the students based on their availability. The walks often took place before or after clinical duties, or during participants’ personal time. The participants decided when the walks ended.

The post-clerkship interviews, which ranged between 40 and 75 minutes, took place immediately following the completion of the LIC to understand participants’ consolidated perspectives regarding their description of the supportive behaviours they experienced throughout their clerkship.

### Data analysis

The interviews were audio recorded and transcribed. Data collection and analyses were iterative, co- constructed with the participants, and reflexive.^32,35^ By taking comprehensive field notes and reflexive journal writing^38-40^, the first author documented audit trails^41,42^ throughout the study in order to enhance the authenticity of the data.

The six steps of inductive thematic analysis proposed by Braun and Clarke informed the process of data analysis.^43^ The first step comprised TD reading each transcript several times to become further familiarized with the data. The second step involved TD completing the preliminary coding of the narratives by making relevant notes about the content and essence for each code throughout each transcript. Step three entailed TD aggregating the coded narratives related to each potential theme. Step four consisted of TD and RJS reviewing the previous steps in relation to the entire data set followed by a data-driven classification of themes and sub-themes developed along with the participants. The methodological emphasis for social constructivist research is on the participant and researcher making meanings together.^32^ The participants played a significant role throughout the interpretation process, particularly in attributing contextual meanings of the narratives. During face-to-face meetings with individual participants, TD made short presentations of the anonymized findings to elicit their reactions and comments to co-construct meaningful interpretations. Participants’ comments and suggestions were integrated to provide additional perspectives in relation to the overall interpretations and presentation of the findings. The fifth step involved ongoing peer review feedback from the participants and the co-authors regarding the overall analysis and definitions for each theme. The sixth step was a detailed analysis and the presentation of the results reported in the present article.

## Results

Data reported in the current paper describe the supportive behaviours the students experienced with a range of individuals who contributed to enhancing their student life during the clerkship year. These key relationships were with: (a) preceptors, (b) peers, (c) family, (d) health professionals, and (e) community members.

### Preceptors

Preceptors are the physicians who taught, supervised, and assessed the medical students’ academic and clinical performances. In terms of the physician-student relationships, the participants’ comments clustered around three sub-themes: (a) physician-student dynamics, (b) physicians as teachers, and (c) physicians as role models.

***Physician-student dynamics*.** Through their longitudinal interactions with preceptors, the participants described being able to learn more about a physician’s role within the medical profession and other aspects of a physician’s life such as assuming different clinical responsibilities, engaging in recreational activities, and being in a family, “You get to know them as individuals…when you’re trying to figure out where you’re going to fit in and what role you want to have in the medical community.” [MS5- during] By the end of clerkship, the participants felt that they were less of a burden on the physician’s workload since they had been able to demonstrate increased confidence and competence with patient care, which they attributed to the mutual trust developed over time. The interpersonal relationships with physicians had a profound effect on the participants’ experiences with becoming a member of the medical community.

***Physicians*** ***as teachers*.** The educational and clinical support from physicians played a substantial role early in the clerkship year, particularly when the participants described being down on themselves for their learning gaps in knowledge and skills. The following participant described developing adaptive strategies regarding clinical approaches by observing his preceptors, “Learning the tricks of the trade and the clinical pearls and the things that come from years of experience.” [MS7-during] Participants described how they felt about the physicians’ willingness to take the time to teach them key concepts and skills throughout their clerkship. They underscored that continuity with the preceptors was important to foster a supportive learning environment.

***Physicians as role models*.** Physician role modeling was critical for learning and support. The participants described how physicians had their own preferred clinical and patient management approaches. One participant reflected on the processes of adaptation they developed in response to the different approaches, “There’s the academic medicine and then there’s the practical medicine.” [MS3-during] The participants discussed the significance that role modeling - resulting from observing several physician-patient interactions - played on how they saw themselves developing as physicians. For example, the following participant described the enriched longitudinal learning as a result of the increased support from physicians in relation to patient-centred care, “It really showed me that working with the patient to create a partnership in their health is going to mean a better result. That’s the kind of physician I want to be.” [MS7-post]

Although there was largely a positive nature to physician role modeling, some participants expressed examples when they observed what they believed to be negative or unsupportive behaviours. For example, one participant shared an experience observing a physician complete a procedure on a patient in labour. The participant explained how troubling the encounter had been for her in the sense that the physician froze the patient without discussing the procedure with her beforehand or obtaining consent. This patient encounter served as a significant moment for the participant in terms of consolidating the impact of the negative role modeling and the learning she was able to take away from the experience.

Even though I’m opposed to that, he’s still a valuable person to be around because he probably does more episiotomies than average… so spending time with him you would learn that specific skill in the event of any critical incident where the baby needs to come out right now because it’s life or death. I tried to rationalize our discussion but I never did get the chance to talk to him about that. That had a major impact on me for sure. [MS2-post]

Other participants described receiving comments about particular areas of medicine as though to discourage them from pursuing certain career paths. The following participant described one encounter when a physician commented unfavourably when she asked to explore psychiatry as a potential career path, “I really like psychiatry so I asked ‘can I do one in psychiatry?’ and the doctor reacted, ‘pfff why would you want to do psychiatry?’ I guess we’re expected to just be content.” [MS8-during] Her experience reflected in part the negative role modeling associated with students gaining exposure to preferred disciplines. The participants described playing an active role in the pursuit of additional learning opportunities that aligned most with their career interests regardless of comments made by preceptors about other specialties.

### Peers

The participants underscored the importance of belonging to a peer group as they established strong ties as a formal social group. The participants’ comments regarding their relationships with peers were grouped together under two sub-themes: (a) peers within the same community and (b) peers at other communities.

***Peers within the same community*.** The participants described feelings of shared social reality support from their peers, which referred to sharing similar experiences with others who can also serve as reality touchstones. The participants recognized that when they reached out to their peers, there was likelihood they shared many of the same challenges. Peers within the community were helpful with developing effective collaborative learning strategies. For example, one collaborative strategy described by the following participant involved creative ways to practicing clinical skills in preparation for an upcoming examination, “We had a little party at one girl’s house and we got pork hocks from the store and practiced our suturing skills.” [MS3-during] Social cohesion was instrumental in formalizing students’ peer social group in their communities and supporting each other, “You build a lot of trust and a lot of expectations between each other and it really worked out.” [MS11-post] The participants shared the importance of socializing as a group and engaging in recreational activity for the purpose of work-life balance. Getting together with peers offered the students with opportunities to wind down and take a break from their busy training requirements.

The participants described the critical reflection they engaged in with their peers, and how they were able to help each other overcome the challenges of the clerkship, “Like coaching one another on how to get what you want out of the clinical, and how to stand up for yourself, that you’re not going crazy, that your thoughts are not irrational.” [MS8-post] The following participant expressed how her relationships with peers in the community evolved during clerkship, and as a result the students were very supportive of each other, “Having spent eight months together, we became a family. Just being open, crying, being angry, being excited, we shared in each other’s joys and sorrows.” [MS2-post]

***Peers at other communities*.** Living, learning, and working so closely alongside peers for the first time was difficult for some of the participants. Therefore, many students initially gained support from having close friends within their student peer group who had been placed in other communities. The following participant described how important it was to stay connected and support each other, “I have friends in other communities that I talk to all the time when I have a bad day or something happens and you need to decompress you know somebody outside of the circle.” [MS3-during] Seeking support from peers outside of the circle was also related to the fact that the participants had previously established study groups and social circles with others from the main campus.

### Family

The participants identified members of their families who were particularly supportive as they adapted to the clerkship such as significant others, siblings, parents, and children. The participants shared how their partners and family members were always available as somebody to turn to for (a) listening and emotional support and (b) onsite support.

***Listening and emotional support*.** Many of the participants shared feelings of loneliness which developed after they relocated to their clerkship community, and away from family and friends. The participants commented on the support they received from their partners and family, often at a distance, in terms of being there for them during the ebbs and flows throughout the clerkship and acting as a sounding board. At the start of clerkship, they described the challenges of integrating into new environments whilst transitioning from the classroom to the clinic, and with their physical displacement from established social support such as friends and family. The participants felt as though their significant others and family provided advice and helped them cope with some of the obstacles associated with living away from home. For example, one participant shared how having a partner who is unfamiliar with medicine was essential in providing her with a different viewpoint on life situations, “It’s nice having someone who can be there just to let you vent about the things that are frustrating you.” [MS9-pre] When things got particularly challenging about two to three months into the clerkship, the participants described the important supportive roles that family played to help them deal with the academic and clinical pressures. The following participant discussed her experience as a parent who had to move away from her husband and children for the clerkship year. She expressed the significant role her family played in supporting her, particularly when it was too difficult to visit.

I wouldn’t be able to do this if I did not have supports. I have a terrific husband, I have great kids, and my extended family is superb. I’m [in the community] at least five days a week. This past weekend was my first weekend home in November, so that’s three weeks without seeing them. I mean I’d drive there for the night, sleep there and come back, but this was my first weekend where I could spend more than eight hours to 12 hours with my family. [MS2-during]

***Onsite support*.** Several of the participants described the benefit of having sources of family support present in the community such as parents, siblings, and significant others, and how helpful it was with reducing some of the unease of undertaking the clerkship. The participants also discussed the importance of onsite family support early in the clerkship given the upheaval being physically displaced, compounded by having to get familiarized with all new surroundings. For example, participants underscored the importance of having family help them with moving their belongings to the community before the start of clerkship, as well as periodic visits from their partners and family throughout clerkship. However, not all participants were in unfamiliar contexts, two of the participants returned to their hometowns and two others to communities with close ties to their families or near their hometowns. The following participant lived at home with her parents and shared how inspired she felt by her parents’ encouragement during her clerkship, “I feel like I’m not the one who’s in medical school, my family is.” [MS11-during] Other participants described the benefit of having their spouse’s staying in the community with them for the entire clerkship, which was particularly important since they had lived away from each other throughout the first two years of medical school.

### Health professionals

The participants described the support from health professionals in addition to physicians. Specifically, the participants referred to the encouragement they received in terms of (a) health professionals as teachers and (b) interprofessional collaboration.

***Health professionals as teachers*.** The participants discussed the clinical support they received from health professionals such as nurses, physician assistants, occupational therapists, and laboratory technologists. Many educational opportunities occurred throughout the clerkship, as described by this participant, “They would go out of their way to provide us with opportunities to practice our skills. They were very supportive. Maybe you wouldn’t get that as much in a larger centre.” [MS3-during] Another participant elaborated on receiving clinical support from a physician assistant, “The physician assistant we met downstairs, he works at the fracture clinic and he taught me how to put on casts. He is a really good teacher and really approachable.” [MS6- during] The participants were very grateful for the supportive behaviours from health professionals besides physicians who helped them learn about the different aspects of medicine.

***Interprofessional collaboration*.** The participants described the advantages of working together with other health professionals in a collaborative team environment. They described the benefits of interprofessional collaboration as they developed a deeper understanding of healthcare service delivery in the clerkship communities. For example, the following participant shared that the longitudinal nature of the clinical training offered more continuity and contributed to making the learning from various health professionals such as nurses more accessible, “The nurses are more constant because there are fewer of them. I was familiar with them. I could ask them things and that would provide some consistency.” [MS9-during] Another participant described how the interprofessional collaboration she experienced changed her views regarding the ways healthcare providers can work together and share expertise, “It’s important to understand people’s health professional roles and where they fit.” [MS4-post] The participants discussed how the support they received from the health professionals supported their competency development and contributed to their sense of belonging to the medical community.

### Community members

The broader community was identified as being essential to the participants’ development of a sense of belonging. The participants’ comments regarding their relationships with the broader community were grouped in three sub-themes: (a) welcoming, (b) integration, and (c) farewell.

***Welcoming*.** The participants lauded the support they received from the community at the start of their clerkship. The following participant described the community members who were involved with the welcome activities and the local media who interviewed her, “We spent a day in the community where we met the mayor, people on the council, people involved in the community, local business owners, banks, they all came out.” [MS6-pre] The participants expressed that the orientation organized by every community was vital to familiarize them with situating the clinical and social settings where they were going to spend most of their time.

***Integration*.** The participants’ integration was largely facilitated from the community support and the fact that it was promoted broadly that medical students were completing their clerkship there, and that together the community could contribute to providing a positive learning experience. This led to patients becoming aware when they encountered the students in clinical settings and their willingness to allow the students to participate in their care. The following participant underscored how once a patient was aware the student was from their community, the next question pertained to where they would eventually practice, “They seem to prefer the local girl, like there’s more pride there I think in the patients. It’s interesting to see how this geography component plays a difference with patient interaction.” [MS10-during] Another participant described how these supportive behaviours contributed to his integration into the community, “We do feel as though the community enjoys having us here. Not once have I felt that we aren’t really accepted, that they don’t want the medical students here.” [MS5-during]

***Farewell*.** The participants referred to the completion of the clerkship as a difficult time to say goodbye to individuals in the community. The participants described the uniqueness of the communities’ interest and contributions to providing positive learning experiences. As a result of the longitudinal clerkship, the participants benefitted from having developed friendly acquaintances and established rapport with many people in the community, which will serve them well as future physicians. One of the participants described how her peer group thanked those in the community who were involved with making them feel a part of the community. She shared how she and her colleagues delivered treats and thank you cards to those who contributed to their experience, “We felt so accepted in the community and we appreciated what has happened to us.” [MS3- post]

## Discussion

The traditional, anonymous African-sourced proverb “*it takes a village to raise a child”* has different meanings influenced by sociocultural environments and contextual relevance in regions around the world. The key tenet among them refers to the significance of the connections within a community actively bonding together toward a common goal to help nurture their young. Based on our findings, the proverb can certainly be translated to *it takes a community to train a future physician*, meaning that supportive individuals such as preceptors, peers, family, health professionals, and community members were identified as being integral sources of social support for the medical students whilst they lived, learned, and worked in a community for eight months.

According to Wenger,^44^ “[…] we all belong to communities of practice […] at home, at work, at school.” Individuals, or medical students in our case, voluntarily wish to join the communities of practice (CoP) and therefore are willingly reassessing their own identities in these contexts.^44^ Students participate in CoP and must re-negotiate personal (e.g., social and physical) and professional (e.g., medical communities) identities simultaneously. Daly and colleagues^45^ concluded that medical students must develop a strong sense of belonging to CoP during longitudinal placements including the medical and broader communities. Within their conceptualization of CoP, Lave and Wenger^46^ characterized this process as legitimate peripheral participation whereby, in the context of a LIC, medical students can become engaged participants in the actual practice of their community. The socialization occurs when students begin to establish and understand their own way of moving from peripheral (e.g., newcomers in the community) to active participants in the medical profession and in the broader community where their learning is taking place.

The present study contributes to the medical education literature about CoP present during a LIC. Researchers have shown that continuity with clinical teachers during longitudinal placements is conducive with the development of meaningful relationships, and is an effective way of fostering students’ competency development.^47,48^ Our findings underscore the importance of students’ ongoing active engagement and participation in CoP and the supportive behaviours they seek during their clerkship experiences. Students reported forming bonds with physician colleagues, and receiving frequent feedback and mentoring from them throughout their clerkship. Our study adds new information regarding how students benefitted from the relationships formed through interprofessional practice^29^ and some serendipitous learning such as preparing for surgical procedures in the operating room, putting on casts at the fracture clinic, and providing diabetes education. In addition, the longitudinal nature of clerkship was a contributing factor to students developing what they called “lasting” friendships with peers and establishing rapport with many other individuals such as patients and community members. We extend prior work examining how medical training during a LIC can help shape students’ sense of belonging in CoP and offer tight knit community support^27,45^ through students’ active participation in the medical profession and the local community, as well as their understanding of the importance of developing mutually meaningful interactions with individuals in the community. Further, community members are closely related to the longitudinal aspects of the students’ clerkship experiences.^49^ The communities see the students as their responsibility whilst ensuring positive learning experiences.^49,50^ There are benefits for the communities in northern and rural regions – they are striving to recruit future physicians to fill local workforce shortages. Indeed, rural-based education clinical experiences for medical learners have been demonstrated as an effective means of training and retaining graduates in rural communities.^51-56^

### Limitations

Although our findings provide unique contributions to the literature, there are limitations to the study. First, the transferability of the findings may or may not be applicable in different learning contexts such as block rotations or a longitudinal placement in an urban context. However, the findings offer insights regarding orientation activities for clerkship students to increase their awareness of social support resources they might reach out to throughout their clerkship experiences. Next, the mean age of the participants in this study is higher than it would be in most contexts. It is possible that those who participated in the study were highly self-directed and self-motivated learners who were actively engaged in the community and participated effectively in educational activities. Participants in our study were predominately female (10 females, two males), which might limit the transferability of our findings to other LIC contexts. Social support may differ based on age and gender. Finally, we did not use social network research methods to examine how the relationships between the students and the sources of social support influenced one another; however, this could represent an area for future research.

### Conclusion

Our findings underscore the important aspects of the relationships between medical students and the sources of social support they seek out during a rural- based LIC. The students experienced social support from various individuals such as health professionals, peers, family, and community members which helped them to manage the demands and challenges of their clerkship more effectively. Medical schools and communities offering longitudinal placements such as a LIC could learn from the students’ experiences with community integration and help foster supportive environments where students live, learn, and work. Students who are better informed about the benefits of supportive behaviours may be able to give more attention to patient care, competency development, and learn how to participate in, and engage with, communities of practice.^57^ Formal orientation activities that prepare students for the type of clerkship experience they are about to embark on can serve to augment reciprocal interpersonal relationships between students and the various supportive resources during longitudinal placements. Our study lends credence to the view that *it really does take a community to train a future physician*.
